# The relationship between race and pain knowledge and attitudes among rural old adults in Alabama

**DOI:** 10.1093/geroni/igaf122.3378

**Published:** 2025-12-31

**Authors:** Lewis Lee, Hyunjin Noh, Haelim Jeong, Lauryn Emrick, Madison Hasnani

**Affiliations:** The University of Alabama, Tuscaloosa, Alabama, United States; The University of Alabama, Tuscaloosa, Alabama, United States; The University of Alabama, Tuscaloosa, Alabama, United States; The University of Alabama, Tuscaloosa, Alabama, United States; The University of Alabama, Tuscaloosa, Alabama, United States

## Abstract

The literature highlights persistent racial disparities in pain assessment and diagnosis, suggesting that marginalized racial and ethnic groups may have limited access to pain education due to systemic inequities in healthcare and education systems. However, little research has explored the relationship between race and pain knowledge and attitudes, particularly in rural Alabama. This study aims to address this gap by examining the level of pain knowledge and attitudes among rural residents and assessing the extent to which race is associated with these outcomes, controlling for covariates. Using purposeful sampling, 123 participants were recruited. Eligibility criteria included being 65 years or older, living outside nursing homes, being cognitively intact, having two or more chronic illnesses and chronic pain, and taking medications for these conditions, including pain medications. The dependent variable was a composite score derived from summing items in the Knowledge and Attitudes Survey Regarding Pain (Cronbach’s alpha=.70). The main independent variable was binary race, and covariates included socio-demographic and health factors, such as the number of chronic health conditions, and pain scores (Cronbach’s alpha=.64). A multiple linear regression model with heteroskedasticity-robust standard errors was used for analysis. Results revealed that BIPOC individuals had significantly lower pain knowledge and attitudes scores compared to their White counterparts (p<.05). Additionally, higher levels of education were positively associated with pain knowledge and attitudes scores (p<.05). These findings emphasize the need to address racial disparities in pain knowledge and attitudes and highlight education’s role in improving perceptions and understanding of pain, particularly among BIPOC populations.

